# Transient changes in L-arginine, asymmetric and symmetric dimethyl arginine in triathletes following Norseman Xtreme Triathlon

**DOI:** 10.3389/fphys.2024.1451038

**Published:** 2024-10-18

**Authors:** Martin Bonnevie-Svendsen, Christoffer Nyborg, Vibeke Bratseth, Jørgen Melau, Jonny Hisdal

**Affiliations:** ^1^ Department of Clinical Medicine, Faculty of Medicine, University of Oslo, Oslo, Norway; ^2^ Department of Vascular Surgery, Section of Vascular Investigations, Aker, Oslo University Hospital, Oslo, Norway; ^3^ Oslo Center for Clinical Heart Research-Laboratory, Oslo University Hospital, Department of Cardiology, Oslo, Norway; ^4^ Joint Medical Services, Norwegian Armed Forces, Sessvollmoen, Norway

**Keywords:** triathlon, NO, endothelium, atherosclerosis, l-arginine, ADMA, SDMA

## Abstract

Arterial vasodilation is dependent on nitric oxide synthesized from L-arginine by endothelial nitric oxide synthase. Triathletes are reported to display altered serum concentrations of nitric oxide metabolites such as L-arginine, asymmetric dimethyl arginine (ADMA) and symmetric dimethyl arginine (SDMA) shortly after completing long-distance triathlon races. In other populations, similar changes to nitric oxide metabolites are established risk markers of cardiovascular disease. The objective of this study was to assess serum concentrations of metabolites for endothelial nitric oxide synthesis in triathletes one week following a long-distance triathlon race. In this prospective observational study, we used high-performance liquid chromatography to measure circulating concentrations of L-arginine, ADMA, and SDMA in triathletes. Venous blood samples were collected before, immediately after, day one, and one week following the triathlon race. Serum concentrations and L-arginine/ADMA ratio were determined for each time-point and compared to baseline. L-arginine/ADMA ratio was reduced on day one (147 ± 32 vs 163 ± 40, *p* < 0.02). ADMA was reduced immediately after and increased at day one and remained elevated at one week (0.29 ± 0.05 μM, *p* < 0.001, 0.44 ± 0.08 μM, *p* < 0.001 and 0.42 ± 0.07 μM, *p* = 0.04, respectively vs 0.40 ± 0.05 μM). SDMA was increased at all time-points when compared to baseline (0.48 ± 0.10 μM, *p* < 0.001, 0.53 ± 0.11 μM, *p* < 0.001 and 0.42 ± 0.08 μM, *p* = 0.048 vs 0.38 ± 0.05 μM). L-arginine was only decreased immediately after (46.0 ± 9.3 μM vs. 64.6 ± 16.1 μM, *p* < 0.001). Long-distance triathlon racing induces altered levels of metabolites for endothelial nitric oxide production that mostly normalizes within one week following racing. The clinical relevance of these transient changes has yet to be elucidated in the athletic population.

## 1 Introduction

Long-distance triathlon races provide a compelling window to observe the boundaries of human cardiovascular physiology. The physical stress induced by 3.8 km swimming, 180 km cycling, and 42 km running is evident in the numerous reports of deviations from normal physiology in triathletes. Observations during and after races include altered ventricular function, pulmonary edema, hyperthermia, hypothermia, electrolyte disturbances, and serum biomarkers well outside physiological reference ranges (Claessens et al., 1999; Danz et al., 2016; Hoiseth et al., 2021; Nyborg et al., 2020; Olcina et al., 2019; Pingitore et al., 2011). The Norseman Xtreme Triathlon (NXTRI) is an annual race that offers athletes an added challenge of inhospitable racing conditions. Athletes face water temperatures as low as 14°C, ambient temperatures down to 6°C, an excess of 5,000 m of total elevation gain, and a marathon run that ends at an exposed mountain peak 1880 m above sea level (Melau et al., 2019). The rising popularity of NXTRI and other triathlon events in the last decades aligns with guidelines that recommend physical exercise to prevent cardiovascular disease (CVD) (Bull et al., 2020; Dahler et al., 2014). However, a U-shaped relationship between exercise volume and CVD mortality has been reported (Schnohr et al., 2015). The potential of excessive exercise to induce negative health consequences remains a topic of discussion (Eijsvogels et al., 2016; Isath et al., 2023). Indeed, endurance athletes have been shown to display accelerated coronary artery atherosclerosis (Mohlenkamp et al., 2008). In search of mechanisms that could explain atherosclerosis in athletes, endothelial cells and their production of nitric oxide (NO) are relevant on grounds of their established anti-inflammatory and cardioprotective properties (Botts et al., 2021). Endothelial cells facilitate vasodilation by synthesizing NO from the essential amino acid L-arginine (Palmer et al., 1988; Schmidt et al., 1988). While three different isozymes have been shown to contribute to vascular NO synthesis, endothelial NO synthase (eNOS) is commonly considered the major regulator of NO production (Garcia and Sessa, 2019). Under certain conditions, other proteins and enzymes, such as hemoglobin and xanthine oxidoreductase may generate NO from reduction of nitrite or nitrate (Garcia and Sessa, 2019). In the endothelium, eNOS activity is influenced by the activity of methylated forms of L-arginine: asymmetric dimethyl arginine (ADMA) inhibits NO synthesis by competing for the binding site of L-arginine on eNOS (Vallance and Leiper, 2002; Vallance et al., 1992). Symmetric dimethyl arginine (SDMA) competes with L-arginine for transport across the endothelial cell membrane, and thus may indirectly suppress NO synthesis (Bode-Boger et al., 2006; Strobel et al., 2012). NO synthesis may to some extent be influenced by homoarginine, which is another substrate for NO synthesis by eNOS. Because homoarginine displays a lower K_m_ than L-arginine it has been suggested that elevated levels of homoarginine may inhibit NO synthesis by competing with L-arginine for cellular uptake (Moali et al., 1998). Of clinical relevance, elevated levels of ADMA and SDMA are reported as independent risk factors for CVD and mortality (Schlesinger et al., 2016). Furthermore, it is well established that prolonged strenuous exercise causes a range of physiological changes that may impact endothelial function, including inflammation and altered bioavailability of essential nutrients (Danielsson et al., 2017; Neubauer et al., 2008; Nyborg et al., 2021a; Storsve et al., 2020). Triathletes at NXTRI have previously displayed impaired endothelial cell function, demonstrated by blunted arterial flow-mediated dilation following race completion (Nyborg et al., 2021b). Congruent with this observation, NXTRI participants also display reduced levels of L-arginine as well as elevated levels of ADMA and SDMA after racing (Nyborg et al., 2021a). Collectively, the above observations prompt further investigation into the potential cardiovascular consequences of exercise-induced changes to NO metabolism in athletic populations. There is a paucity of literature on the behavior of NO metabolites beyond the initial 24 h following physical exercise. Therefore, the purpose of this study is to assess the serum concentrations of L-arginine, ADMA, SDMA, and L-arginine/ADMA ratio up to one week after completion of a triathlon competition.

## 2 Methods

### 2.1 Design and study population

In this prospective observational study, we recruited adult triathletes partaking in the annual NXTRI during 2019–2022. Invitation to voluntarily participate in the study was issued via email to all athletes (n = 578) registered for the NXTRI via email and on the race organizer’s website in the period July to August year 2019, 2021, and 2022. All athletes above the age of 18 who could attend the planned one-week follow-up examination were included (n = 25). Exclusion criteria included the presence of established kidney or liver disease. Subjects were asked to report on past medical history via a questionnaire issued within 48 h before the race. This included questions on existing medical disorders, drug use, and timing of menstrual cycle. A note was made of subjects who reported existing cardiovascular or metabolic disease and their results were assessed for outliers. The study was approved by the Regional Committee for Medical and Health Research Ethics in Norway (REK Sør-Øst 2016/932 and 481,115) and was conducted per the declaration of Helsinki. All participants were required to give written informed consent before being included in the study.

### 2.2 Blood sampling and laboratory analyses

Venous blood samples were collected at four time-points: within 48 h before the race start (baseline), within 60 min after the race finish (immediately after), at noon the day after completing the race (day one), and six to nine days after the race (one week). The blood samples were drawn from an antecubital vein into vacutainers containing silica particles and gel separators and allowed to clot at room temperature for 30 min. They were further centrifuged at 2000 *g* for 10 min to make serum. Serum was pipetted and transferred to freeze-resistant vials and then refrigerated. We transported the refrigerated samples to Oslo University Hospital (Aker Sykehus, Oslo, Norway) for storage at −80 °C in the hospital biobank until analysis. Analyses were performed November 2023 at Oslo University Hospital (Ullevål Sykehus, Oslo, Norway) by high-performance liquid chromatography and pre-column derivatization with o-phtaldialdehyde (Sigma Chemicals CO, St Louis, MO, USA) for levels of L-arginine, ADMA and SDMA (inter-assay coefficients of variation: L-arginine 5.9%; ADMA 7.0%; SDMA 6.6%).

### 2.3 Data management and statistics

Data for each test variable were plotted for visual assessment of distribution. One data point for SDMA in a single subject was deemed unphysiological and discarded from the analysis as a measurement error. One-way repeated measures ANOVA with time points as a within-subjects factor was conducted for L-arginine, ADMA, SDMA, and L-arginine/ADMA ratio at all four time points. Post-hoc paired t-tests were performed to compare each time point against the baseline. We applied the Holm-Sidak method to correct for multiple comparisons. Statistical significance was set to *p*-values < 0.05. Results are given as means ± SD. All statistical analyses and plots were created in SigmaPlot (version 15.0.0.13, Inpixon, Palo Alto, California, USA).

## 3 Results

### 3.1 Subjects

25 participants (male = 18, female = 7) from the 2019, 2021 and 2022 edition of NXTRI were included in the study. Two subjects failed to complete the race, and a further two failed to present to examination at one week follow-up due to illness and unknown reasons. 21 subjects (male = 16, female = 5) completed the race and attended testing at all time points. The subject characteristics are presented in Table 1.

**TABLE 1:** Subject characteristics.

	Mean ± SD
Age (yr)	40.4 ± 9.8
Race seasons completed (yr)	6.8 ± 3.6
Full-distance triathlons completed	7.0 ± 10.2
Body mass (kg)	74.6 ± 9.7
Height (m)	1.78 ± 0.07
BMI	23.4 ± 4.7
Weekly endurance training (h)	14.4 ± 4.7
Weekly resistance training (h)	1.0 ± 0.8
NXTRI swim time (h)	1.4 ± 0.4
NXTRI cycling time (h)	7.0 ± 1.2
NXTRI run time (h)	5.4 ± 1.2
NXTRI finish time (h)	14.0 ± 2.5

### 3.2 Laboratory analyses of NO metabolites

**FIGURE 1:**
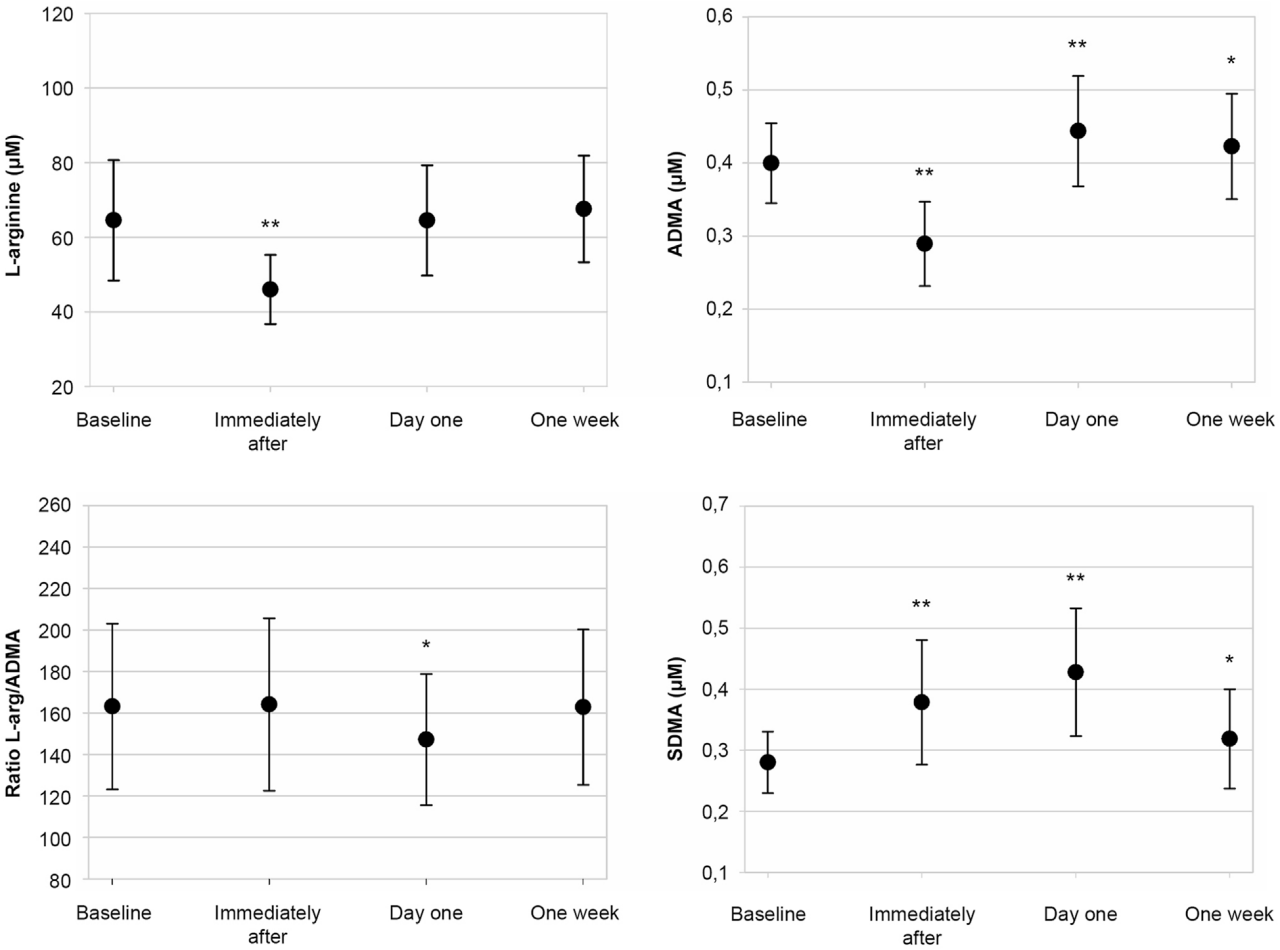
Scatter plot of serum concentration of NO metabolites. Mean serum concentrations and standard deviation at baseline, immediately after, day one, and 1 week. Statistically significant difference from baseline is illustrated with * (*p* < 0.05) and ** (*p* < 0.001).

**FIGURE 2:**
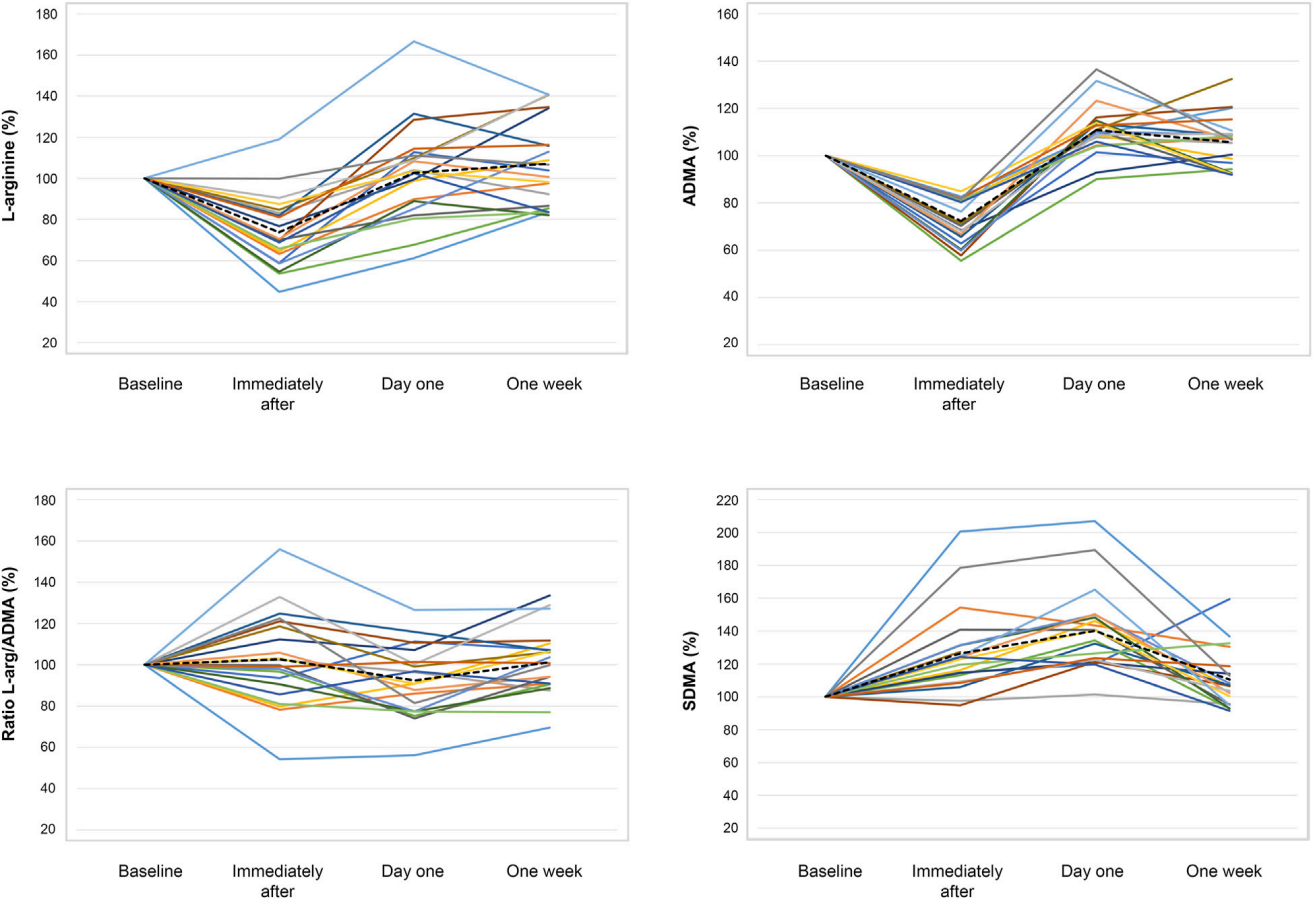
Changes to individual subject serum concentrations. Serum concentrations are given in % of the concentration at baseline for each subject. The mean of all samples at a given time is illustrated by the dotted line.

Mean values and standard deviation for L-arginine, ADMA, SDMA, and L-arginine/ADMA ratio are presented in Figure 1. L-arginine decreased markedly from baseline (64.6 ± 16.1 μM) to immediately after (46.0 ± 9.3 μM, *p* < 0.001) with no difference from baseline at later time points. Similarly, ADMA was reduced from baseline (0.40 ± 0.05 μM) to immediately after (0.29 ± 0.05 μM, *p* < 0.001) before increasing at day one (0.44 ± 0.08 μM, *p* < 0.001) and remaining elevated at one week (0.42 ± 0.07 μM, *p* = 0.04). SDMA displayed an increase from baseline (0.38 ± 0.05 μM) to immediately after (0.48 ± 0.10 μM, *p* < 0.001), with a further increase on day one (0.53 ± 0.11 μM, *p* < 0.001) and numerically higher levels at one week (0.42 ± 0.08 μM, *p* = 0.048). Finally, the L-arginine/ADMA ratio remained unchanged from baseline (163 ± 40) to immediately after (164 ± 42 μM, *p* < 0.001), with a reduction on day one (147 ± 32, *p* < 0.02) and a return towards baseline at one week (163 ± 37, *p* < 0.98). The changes in serum concentrations for each subject at all time points are presented in Figure 2.

## 4 Discussion

### 4.1 Main findings

The main finding in the present study is that triathletes display transient changes in serum concentrations of NO metabolites that mostly normalizes in the week after completing a long-distance triathlon. Specifically, the L-arginine/ADMA ratio was reduced and ADMA and SDMA increased the day after the race, with a return to baseline one week later for all parameters except ADMA, which remained slightly elevated. The reported changes in NO metabolites are congruent with a transient reduction in NO synthesis.

### 4.2 NO metabolites in the general population

To the best of our knowledge, there are no established reference ranges for the NO metabolites examined in this study. When comparing our data to a large sample (n = 3,952) of the general population by Schwedhelm et al. (2014), L-arginine were considerably lower in NXTRI athletes with a mean of 46.0 (±9.3 μM) immediately after the race, compared to a median of 152 (25th; 75th percentile, 120; 188 μM) reported by Schwedhelm et al. The lower L-arginine also means NXTRI athletes displayed a lower L-arginine/ADMA ratio than that calculated from Schwedhelm’s data. Specifically, the L-arginine/ADMA ratio was reduced and ADMA and SDMA increased the day after the race, with a return to baseline one week later for all parameters except ADMA, which remained slightly elevated (Schwedhelm et al., 2014). However, such comparison should be interpreted with some caution as differences in analysis methodology could well contribute to discrepancies.

### 4.3 NO metabolites after exercise

Our results correlate with observations from Nyborg et al. (2021a), who found a similar pattern of depressed L-arginine/ADMA ratio and elevated ADMA and SDMA one day after a past edition of the NXTRI. Furthermore, Mieszkowski et al. (2023) made similar reports of elevated ADMA and SDMA in runners 24 h after completing an ultramarathon race. In contrast to our results, these runners displayed no change in the L-arginine/ADMA ratio. It is possible that this discrepancy could be explained by differences in work duration, exercise intensity, or differences in exercise modalities between triathlon and ultra-marathons. More lenient exercise protocols have shown conflicting results with the studies mentioned above. Riccioni et al. (2015) found elevated plasma L-arginine and L-arginine/ADMA ratio, with depressed ADMA and SDMA concentrations immediately after 10 min of treadmill walking in subjects with coronary artery disease. Similarly, increased L-arginine and decreased ADMA and SDMA concentrations have been reported following cardiopulmonary exercise tests in subjects with chronic heart failure (Drohomirecka et al., 2023). Furthermore, Argunova et al. (2022) oobserved that five to ten days of 40 min of daily treadmill workouts appeared to stabilize ADMA concentrations in patients with coronary artery disease. Collectively, it appears that modest aerobic exercise may promote short-term NO synthesis and endothelial cell function in patients with CVD. Whereas vigorous endurance exercise of prolonged duration appears to shift NO metabolites in a direction that may lead to transiently impaired NO synthesis in athletes.

### 4.4 Proposed mechanisms for altered NO metabolite concentrations

The mechanisms underlying altered levels of L-arginine, ADMA and SDMA after exercise have yet to be fully elucidated. During exercise, cardiac output increases, and blood flow is redistributed to meet the oxygen demand in the working muscles. This redistribution is facilitated by vasodilation in the involved skeletal muscles (Joyner and Casey, 2015). The authors deem it plausible that the decreased L-arginine immediately after finish could be a result of substrate depletion from the prolonged duration of exercise during NXTRI. It has been speculated that homoarginine, which is considered a weaker substrate for NO synthesis, could impact the cellular uptake of L-arginine if the balance between homoarginine and L-arginine is shifted sufficiently in favor of the former (Moali et al., 1998). As we did not measure homoarginine in our sample we cannot conclude on its potential role. Past studies on NXTRI athletes have shown increased creatinine kinase and elevated markers of inflammation post-race (Nyborg et al., 2020). We have therefore argued that post-race increases in ADMA and SDMA in triathletes may be due to increased proteolysis from exercise-induced muscle damage and inflammation (Nyborg et al., 2021a). Furthermore, ADMA and SDMA are both excreted via the kidneys and to a lesser extent cleared by the liver (Siroen et al., 2025). As NXTRI athletes have been shown to display increased serum concentrations of both creatinine and liver enzymes after racing, it could be that reduced clearance by the liver and kidneys contributes to the elevated ADMA and SDMA values (Nyborg et al., 2020).

### 4.5 NO metabolites as prognostic marker for CVD

The consequences of exercise-induced changes in NO metabolism in the athletic population have yet to be elucidated. Elevated ADMA and SDMA have been associated with increased risk of all-cause mortality, cardiovascular disease or worsened prognosis in a range of conditions and populations. These include heart failure, type 2 diabetes mellitus, critically ill patients, and patients with cardiac arrest (Keller et al., 2020; Lee et al., 2022; Wirth et al., 2017; Zobel et al., 2017). In the general population, higher levels of ADMA are associated with all-cause mortality and CVD, whereas high SDMA is associated with all-cause mortality (Schlesinger et al., 2016). Furthermore, a higher arginine/ADMA ratio has been associated with reduced incidence of cardiovascular events and atherosclerotic development in high-risk and general populations, respectively (Notsu et al., 2015; Yu et al., 2017). To the best of our knowledge, the prognostic value of NO metabolites has yet to be described in the athletic population. Furthermore, it is not yet established whether a transient change in NO metabolites post-exercise carries the same prognostic value as altered baseline values. It is prudent to consider that several established risk factors for CVD, such as cigarette smoking, hypertension, and dyslipidemia affect risk in a dose-dependent manner (Bhat et al., 2008; Zhang et al., 2019). If exercise-induced changes to NO metabolites were indeed predictive of CVD in triathletes, it would be logical to assume their impact is dose-dependent. It is our experience, that the duration of long-distance triathlon races far exceeds the duration of workouts triathletes undertake in regular training. Importantly, triathletes train far more often than they race. We, therefore, suggest that future studies should seek to explore the behavior of NO metabolites in triathletes after their day-to-day exercise. Furthermore, prospective studies with longer follow-up duration could help clarify whether exercise-induced changes in NO metabolites translate to altered risk for CVD or if these changes represent benign physiological adaptations.

### 4.6 Strengths and shortcomings

The present study provides descriptive data on NO metabolites in triathletes following workloads that would typically be considered impractical for testing in controlled laboratory conditions. This allows insight into physiological responses that would otherwise remain unexplored. The novelty of the present study is the extended follow-up period beyond what has previously been reported in the athletic population. Nevertheless, the results should be interpreted with some caution. Of note, we only measured concentrations of L-arginine, ADMA, and SDMA in serum, whereas the synthesis of NO by eNOS occurs intracellularly. Extracellular concentrations of NO metabolites may not proportionally reflect the activity of intracellular eNOS activity. However, the present results align with past observations of transiently reduced vascular responsiveness following the NXTRI, as expressed via lowered flow-mediated dilation with concomitantly reduced L-arginine and elevated markers of inflammation (Nyborg et al., 2021b; Nyborg et al., 2021a). We therefore consider it plausible that the changes to L-arginine/ADMA ratio, ADMA, and SDMA of 9.0, 11.0, and 38.9% might contribute to lowered NO synthesis and blunted end-organ activity. The participants in this study were not screened for CVD other than by a questionnaire. While all but three subjects reported no history of CVD or pre-existing conditions with known impact on NO precursors, we cannot entirely rule out that undetected medical conditions may have influenced our results. The results are therefore best regarded as an observation of NO metabolite behavior in triathletes irrespective of any conditions they may or may not have. While this could be considered a methodological weakness, the fact remains that altered NO metabolites have shown prognostic value in both healthy and diseased populations (Keller et al., 2020; Schlesinger et al., 2016; Yu et al., 2017). We therefore argue that the current observations are still worthwhile consideration.

## 5 Summary

This study demonstrates transient changes in serum L-arginine/ADMA ratio, SDMA, and ADMA concentrations in the week following a long-distance triathlon. Deviations from baseline serum concentration mostly normalized within one week, except for ADMA which remained slightly elevated. These changes are consistent with temporarily reduced substrate availability for endothelial NO production and could be an expression of transiently suppressed endothelial cell function. Future studies should seek to elucidate whether exercise-induced changes to NO metabolites are a benign physiological phenomenon or one that contributes to the risk of future CVD.

## Data Availability

The original contributions presented in the study are included in the article/Supplementary Material, further inquiries can be directed to the corresponding author.

## Abbreviations

ADMA, Asymmetric dimethyl arginine; CVD, Cardiovascular disease; eNOS, Endothelial nitric oxide synthase; NO, Nitric oxide; NXTRI, Norseman Xtreme Triathlon; SDMA, Symmetric dimethyl arginine.
